# Imaging Mass Spectrometry Visualizes Ceramides and the Pathogenesis of Dorfman-Chanarin Syndrome Due to Ceramide Metabolic Abnormality in the Skin

**DOI:** 10.1371/journal.pone.0049519

**Published:** 2012-11-15

**Authors:** Naoko Goto-Inoue, Takahiro Hayasaka, Nobuhiro Zaima, Kimiko Nakajima, Walter M. Holleran, Shigetoshi Sano, Yoshikazu Uchida, Mitsutoshi Setou

**Affiliations:** 1 Department of Cell Biology and Anatomy, Hamamatsu University School of Medicine, 1-20-1 Handayama, Higashi-ku, Hamamatsu, Shizuoka, Japan; 2 Department of Applied Biological Chemistry, Kinki University, Nara, Nara, Japan; 3 Department of Dermatology, Kochi Medical School, Kochi University, Kohasu, Okocho, Nankoku, Nankoku, Japan; 4 Department of Dermatology, School of Medicine, University of California San Francisco, Department of Veterans Affairs Medical Center, and Northern California Institute for Research and Education, San Francisco, California, United States of America; MUSC SC College of Pharmacy, United States of America

## Abstract

Imaging mass spectrometry (IMS) is a useful cutting edge technology used to investigate the distribution of biomolecules such as drugs and metabolites, as well as to identify molecular species in tissues and cells without labeling. To protect against excess water loss that is essential for survival in a terrestrial environment, mammalian skin possesses a competent permeability barrier in the stratum corneum (SC), the outermost layer of the epidermis. The key lipids constituting this barrier in the SC are the ceramides (Cers) comprising of a heterogeneous molecular species. Alterations in Cer composition have been reported in several skin diseases that display abnormalities in the epidermal permeability barrier function. Not only the amounts of different Cers, but also their localizations are critical for the barrier function. We have employed our new imaging system, capable of high-lateral-resolution IMS with an atmospheric-pressure ionization source, to directly visualize the distribution of Cers. Moreover, we show an ichthyotic disease pathogenesis due to abnormal Cer metabolism in Dorfman–Chanarin syndrome, a neutral lipid storage disorder with ichthyosis in human skin, demonstrating that IMS is a novel diagnostic approach for assessing lipid abnormalities in clinical setting, as well as for investigating physiological roles of lipids in cells/tissues.

## Introduction

Imaging mass spectrometry (IMS) has several advantages for exploring the two-dimensional distribution of lipids [1]–[3]: First, IMS does not require any labels or specific probes to investigate the localization; second, IMS is a non-targeted imaging method, allowing us to detect the localization of unexpected metabolites [4]–[7]. Finally, IMS allows the simultaneous imaging of many types of molecular species at once. Therefore, IMS has been a powerful technique for characterizing and/or determining the distribution of molecular species on tissue sections [8], [9]. IMS has been applied to lipid imaging analysis because lipids have relatively small molecular sizes compared to proteins/peptides and lack specific probes for other imaging techniques. A technical difficulty of IMS exists in the ionization efficiency of some lipids, including ceramides (Cers). In addition, the resolution and sensitivities of IMS are based on laser and ionization methods. The recent experimental model of IMS demonstrates the highest resolution (<1 µm) [10], while conventional matrix-assisted laser desorption/ionization (MALDI) imaging mass spectrometers are equipped with lasers of diameter 10–100 µm [11]–[14]. We have developed the instrument composed of an atmospheric-pressure (AP) ion-source chamber for MALDI and a quadrupole ion trap time-of-flight (QIT-TOF) mass spectrometer [15]. Using a 10-µm-diameter laser, the instrument can visualize the distribution of biomolecules, including volatile molecules. Since this machine employs an AP ion-source chamber that utilizes soft ionization and a QIT that concentrates the specific ions to be analyzed, we hypothesize that ion suppression of Cers from other lipids is avoided in this case.

**Figure 1:**
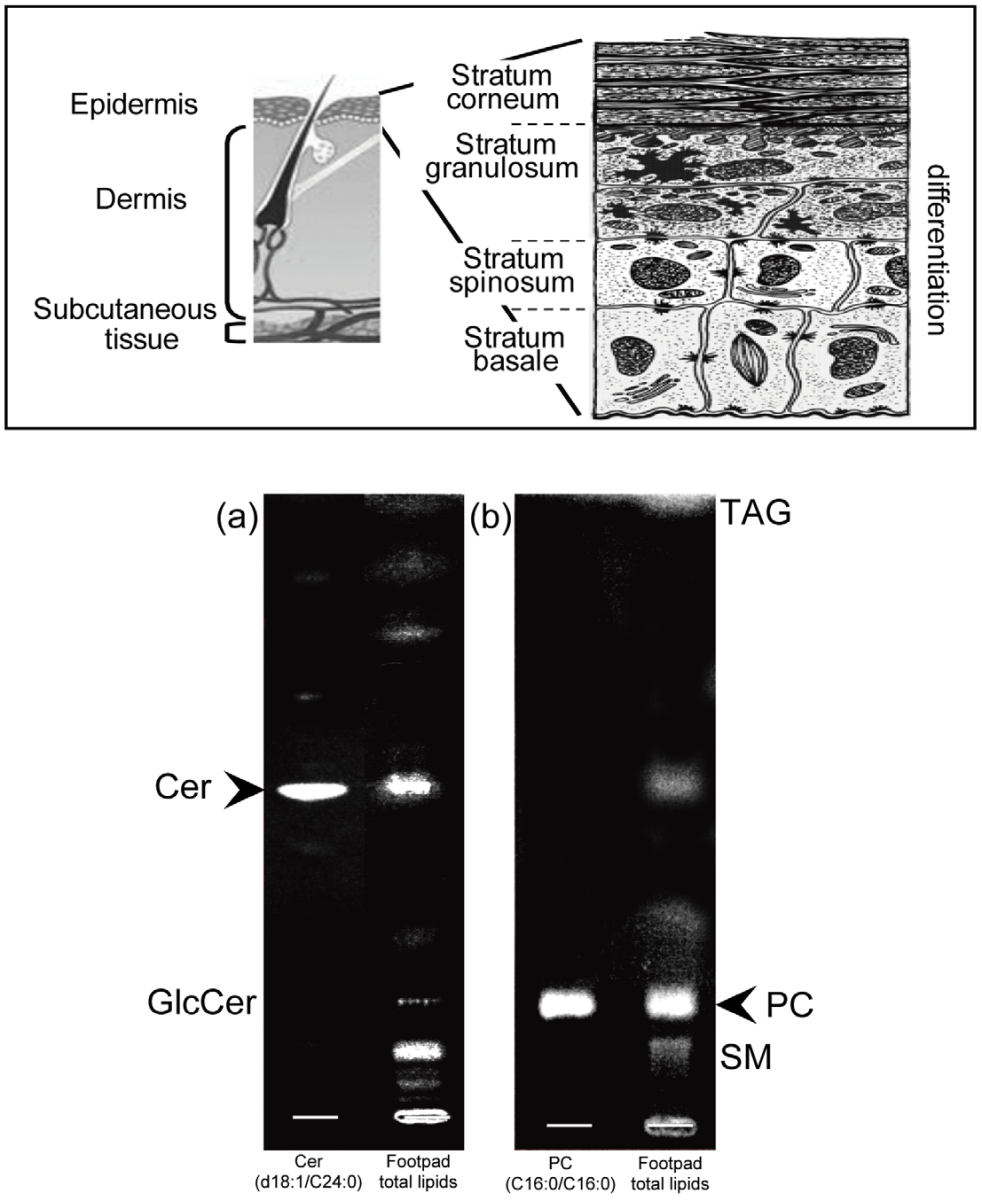
Skin structure and lipid composition of skin. Upper panel shows a model of the structure of the skin. Lower panel shows the results of thin-layer chromatographic analyses of lipid in mouse skin. We assessed lipid composition enriched in mouse footpad skin by TLC analysis. Cer and PC are shown as major lipid species. In addition we also detected GlcCer (a), SM, and TAG (b).The arrowhead shows the localization of standard, Cer (d18∶1/C24∶0) and PC (C16∶0/C16∶0).

Mammalian skin possesses a competent barrier to prevent excess water loss, localized in the outer layer of the skin, the epidermis, predominantly consisting of keratinocytes. Keratinocytes proliferate at the stratum basale (SB), and migrate towards the skin surface to the stratum spinosum (SS), the stratum granulosum (SG), and then the stratum corneum (SC), in parallel with their differentiation (Fig. 1). The SC is largely responsible for the barrier function of the skin. This outermost epidermal layer is composed of terminally differentiated (denucleated) keratinocytes, and corneocytes, surrounded by a mixture of lipids (mainly Cers, cholesterol, and fatty acid), which together form continuous multilamellar membranes serving as a permeability barrier.

Cers are particularly unique in the epidermis in displaying molecular heterogeneity. At least ten molecular groups of species exist due to the variation of the sphingosine base and amide-linked fatty acids (Fig. S1). Ceramide-1-phosphate (Cer1P), which is a metabolite of Cer generated by ceramide kinase, is an anionic bioregulatory lipid that translocates and directly activates cytosolic phospholipase A2, making it a leading mediator of inflammatory responses [16]. Yet, the roles of Cer1P in epidermal function are unknown.

Glucosylceramide (GlcCer) is a simple glycosphingolipid composed of one mole each of glucose and Cer. GlcCer is enriched in certain tissues, including mammalian skin, and is the major precursor for more complex glycosphingolipid. During differentiation, the most newly synthesized heterogeneous Cer species in the SG are glucosylated to GlcCer [17] or are phosphocholinated to sphingomyelin (SM), and then most are packed into lamellar bodies [18]. The lamellar body membrane fuses with the plasma membrane on the apical surface of the granular cells, at which point its contents are extruded into the interface between the SG and the SC. In parallel to the transition from SG to SC, the glucose moiety of GlcCer is hydrolyzed by ß- glucocerebrosidase, resulting in the production of Cer in the SC [19]–[21]. A deficiency of ß- glucocerebrosidase or inhibition of its activity in the epidermis decreases the amount of Cer in the SC and results in failure to form a competent epidermal permeability barrier [19], [20]. Sphingomyelinase-mediated hydrolysis of SM also contributes to the barrier’s homeostasis [22], [23]. These prior studies indicate that both GlcCer and SM are immediate precursors for the Cers in the SC. Furthermore, previous studies have demonstrated that not only the total amount of Cers in the SC, but also the distribution of each Cer molecular species is important for the formation of lamellar structures in the SC [24]. Indeed, alteration of Cer composition has been reported in several skin diseases; *i.e.,* atopic dermatitis, psoriasis, and certain ichthyoses that also display epidermal barrier abnormalities [25]–[28]. Little is known, however, about the mechanism(s) responsible for these alterations; *i.e.,* abnormalities of localization and/or synthesis. Thus, characterization of the specific distribution of each Cer species is important in order to elucidate the relationship between Cers in the SC and the barrier defects of those skin diseases.

Attenuation of permeability barrier function, which not only affects skin (atopic dermatitis, psoriasis, and some ichthyoses), but also affects systemic issues (asthma, food allergy, infection) is a pathogenesis in a number of diseases. In addition, barrier abnormalities cause disease phenotype in several inherited cutaneous diseases, including Dorfman-Chanarin syndrome, a focus of this manuscript. Hence, an accurate, precise, and sensitive method for the quantification of Cer is required for not only investigation of the physiological function of distinct Cer, but also for clinical reasons, *i.e.,* diagnosing Cer deficiencies in subjects provides both pathogenesis and potential therapeutic approaches. In addition, patients, their families, and communities need accurate diagnoses for many reasons, including proper therapy, minimization of disease development, and decreased risks of transfering diseases to the next generation. Currently, Cers are analyzed by enzymatic diacylglycerol kinase assay [29], thin-layer chromatography (TLC) detection [30], high-performance liquid chromatography (HPLC) [31], [32], HPLC-mass spectrometry or tandem mass spectrometry [33], [34], and gas chromatography mass spectrometry analysis [35]. These approaches of analyzing Cer levels in skin require large amounts of skin samples. In addition, these methods are not able to localize Cer species in the tissues/cells. Whereas immunoelectron microscopic analysis can localize total Cer species in cells and tissues, it cannot localize each molecular species of Cer. A recent study demonstrated skin lipid analysis of sphingolipids, including SM and Cer1P, but not Cer and GlcCer, using MALDI-IMS (spatial resolution is>30 µm) [36]. We here demonstrate that IMS characterizes the distribution of Cer species, as well as Cer1P, GlcCer, SM, and phosphatidylcholine (PC) using 3–5 mm diameter biopsy samples. In addition, our present study reveals new insights into epidermal lipids directly from mammalian tissues and the epidermal metabolic abnormality of a key Cer species, AcylCer, occurring in Dorfman-Chanarin syndrome, a neutral lipid storage disorder with ichthyosis in human skin.

## Results

### Lipid Composition on Mouse Footpad Skin

Because imaging mass spectra are directly acquired from a section of tissues which contain various amounts of substances, abundant molecules that have higher ionization efficiency are preferentially detected. Therefore, we first assessed lipid composition enriched in murine footpad skin by TLC analysis. The chromatograms showed that Cer (Fig. 1a) and PC (Fig. 1b) are major components. In addition, we also detected GlcCer (Fig 1a), SM, and triacylglycerol (TAG) (Fig 1b). While epidermal Cers comprise a number of heterogeneous species (Fig. S1) [37], one species, Cer (amide-linked non-hydroxy fatty acid and sphingosine) was detected as the major component in mouse footpads, having a similar mobility to the Cer standard (d18∶1/C24∶0).

### The Detection of Ceramide Molecules on Tissue Sections

**Figure 2:**
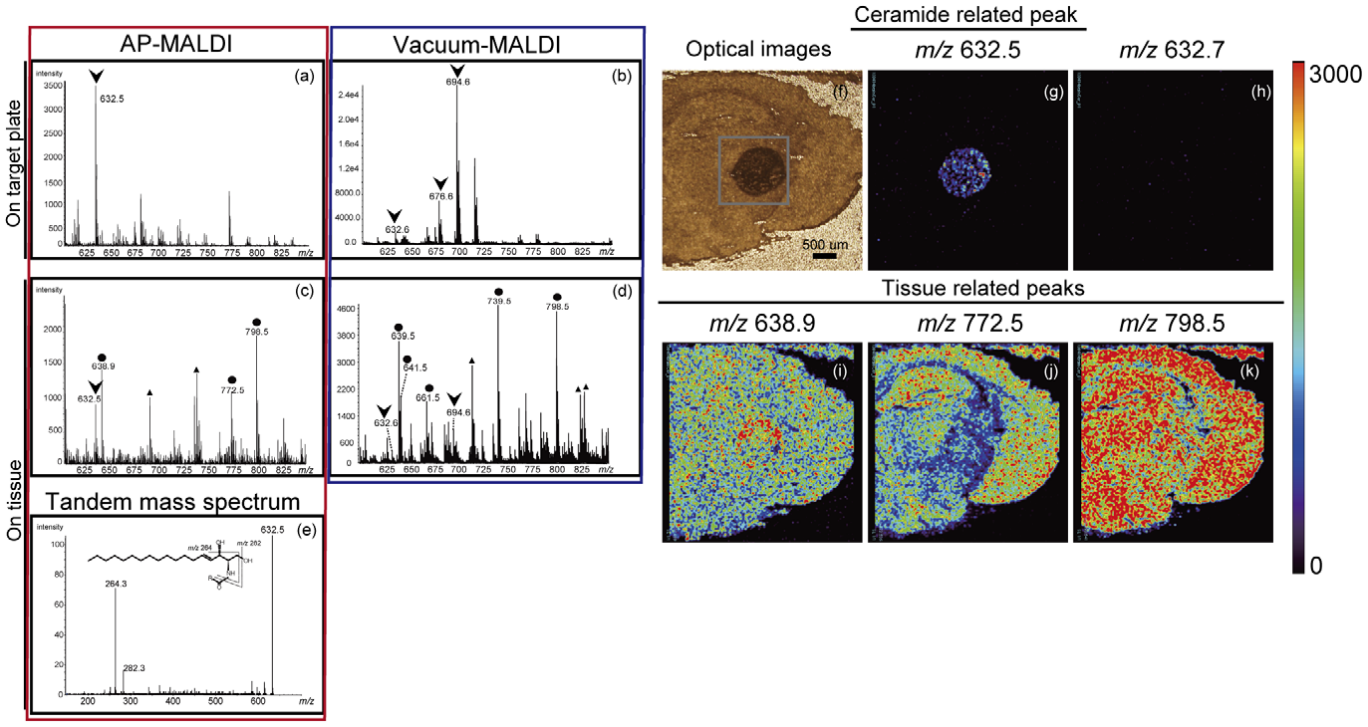
Detection of Ceramides by mass spectrometry. The ionization patterns of Cer standard (d18∶1/C24∶0) deposited on stainless-steel target plates with AP ion-source instrument (a) and vacuum-type instrument (b). The Cer standard (d18∶1/C24∶0) was deposited onto a mouse brain tissue section and the mass spectrum was acquired (c and d). A subsequent tandem mass spectrometric analysis of precursor ions at *m/z* 632.5 was shown (e). The optical image (f) and ion image revealed that *m/z* 632.5 was predominantly derived from exogenous Cer (g). The ion image at *m/z* 632.7 is also shown (h). Moreover, we visualized ion images of major tissue-derived signals (*m/z* 638.9, 772.5, and 798.5) (i–k). Color bar shows signal intensity.

We first investigated the ionization patterns of the Cer standard (d18∶1/C24∶0). Figure 2a shows the mass spectrum of Cer (d18∶1/C24∶0) on stainless target plates with our AP ion-source instrument. We detected *m/z* 632.5 as a dominant peak; it was assigned to the [M−H_2_O+H]^+^ion. We applied the same scheme with the commercial intermediate vacuum-type MALDI instrument. Although signal intensity of Cer-related ions by vacuum-type MALDI instrument was higher than the AP ion-source instrument, there is no significant difference of a signal-to-noise (S/N) ratio between the two instruments. In contrast to AP ion-source instrument that showed single [M−H_2_O+H]^+^ion, multiple adduct ions, such as [M−H2O+H]^+^, [M−H2O+2Na−H]^+^, and [M+2Na−H]^+^of Cer-related ions were detected in the vacuum-type MALDI instrument (Fig. 2b). Similar results were shown in another intermediate vacuum-type MALDI instrument (data not shown). Multiple substances on a tissue section interfere with and diminish each other’s ionization. Therefore, we deposited the Cer standard (d18∶1/C24∶0) onto a mouse brain tissue section and acquired the mass spectrum of gray square area in the optical image (Fig. 2f). A mass signal, [M−H_2_O+H]^+^, was detected from deposited Cer (arrowhead) in addition to endogenous tissue-derived signals (closed circle) (Fig. 2c). On the contrary, a vacuum-type instrument showed various kinds of adduct ions of Cer, and resulted in a complicated spectrum (Fig. 2d). Subsequent tandem mass spectrometric analysis revealed the generation of stable product ions with *m/z* 282.3 and 264.3 corresponding to the loss of an amide-bound acyl group and one or two molecules of water, respectively [33] (Fig. 2e), indicating that the signal at *m/z* 632.5 detected on the brain tissue is a Cer molecule. Moreover, we also visualized ion images of major tissue-derived signals (*m/z* 638.9, 772.5, and 798.5), as well as Cer at *m/z* 632.5 (Fig. 2g) and assigned *m/z* 798.5 as PC (diacyl-16∶0/18∶1) (Fig. S3) as we reported previously [6]. Other molecules, at *m/z* 638.9 and 772.5, were not assigned (Fig. 2i, j, k). To verify mass accuracy, we also constructed an ion image at *m/z* 632.7 (Fig. 2h).

### Imaging Mass Spectrometric Analysis of Ceramide Species in Mouse Footpad Skin

**Figure 3:**
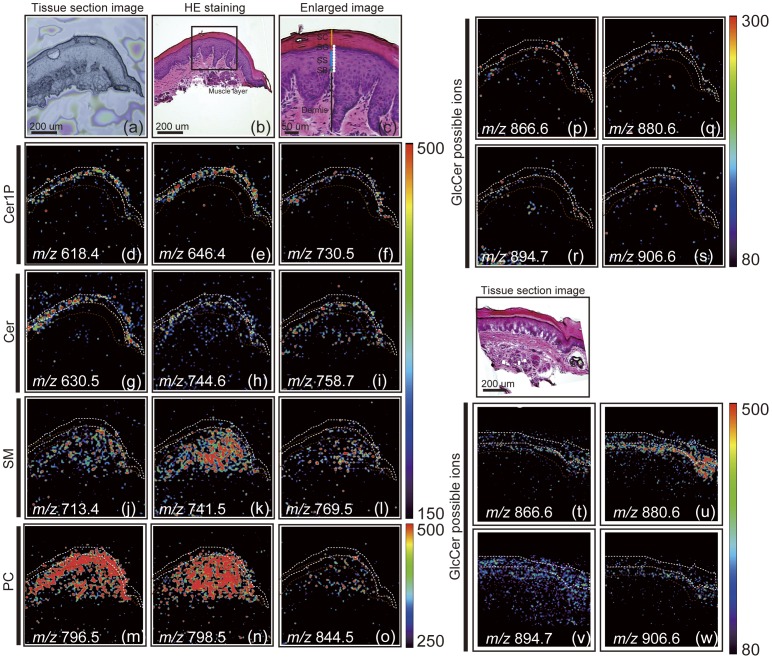
IMS analyses revealed the localization of Cer and Cer-related ions in a mouse footpad skin section. Optical image of mouse footpad section (a), the HE staining image of the serial section (b), the enlarged image (c) are shown. We could detect four layers in the epidermis: SC, SG, SS, and SB. Ion images of Cer1P (d–f), Cer (g–i), SM (j–l), PC (m–o) and GlcCer possible ions (p–s) are shown (SC indicated as a white line; SS and SG indicated as brown lines). We tried to increase sensitivities of GlcCer by limiting detection mass ranges, 800–1200 (t–w). The imaging pixel is 10 µm (a white dot indicates 10 µm, [c]). Color bar shows signal intensity.

We demonstrated here the distribution of various kinds of Cer molecular species on the footpad skin by IMS. The optical image of skin sections, the HE staining images of serial sections and enlarged images are shown in Figures 3a, b, c. IMS analyses, using our AP ion-source instrument, detected some peaks enriched in the fine region of the epidermis. Taken together with tandem mass spectrometric analyses and prior studies that characterize mammalian epidermal Cer species [38], [39], we constructed some ion images detected by IMS, as summarized in Table 1. Three Cer1P (*m/z* 618.40, 646.46, and 730.57) and three Cer peaks (*m/z* 630.50, 744.68, and 758.70) were deduced as Cer1P (d18∶1/C16∶0, d18∶1/C18∶0, d18∶1/C24∶0), and Cer (d18∶1/C24∶1, d18∶1/C32∶0, and d18∶1/C32∶1h), respectively (Figures 3d, e, f, g, h, i). By comparing the fragment patterns of standard Cer (d18∶1/C24∶1) and Cer1P (d18∶1/C18∶0), we successfully confirmed these structures (Fig. S3).

**Table 1:** List of the molecules observed in the mouse footpad.

Observed m/z	Theoletical m/z	tolerance	ion	possible molecular structure
618.4	618.48	0.08	[M+H]^+^	Ceramide-1-phosphate (d18∶1/C16∶0)
630.5	630.62	0.12	[M−H_2_O+H]^+^	Ceramide (d18∶1/C24∶1)
646.46	646.51	0.05	[M+H]^+^	Ceramide-1-phosphate (d18∶1/C18∶0)
713.49	713.49	0	[M+K]^+^	Sphingomyelin (d18∶1/C14∶0)
730.57	730.63	0.06	[M+H]^+^	Ceramide-1-phosphate (d18∶1/C24∶0)
741.51	741.53	0.02	[M+K]^+^	Sphingomyelin (d18∶1/C16∶0)
744.68	744.72	0.04	[M−H_2_O+H]^+^	Ceramide (d18∶1/C32∶0)
758.7	758.72	0.02	[M−H_2_O+H]^+^	Ceramide (d18∶1/C32∶1h)
769.52	769.56	0.04	[M+K]^+^	Sphingomyelin (d18∶1/C18∶0)
796.53	796.52	−0.01	[M+K]^+^	Phosphatidylcholine (C16∶0/C18∶2)
798.55	798.54	−0.01	[M+K]^+^	Phosphatidylcholine (C16∶0/C18∶1)
844.54	844.52	−0.02	[M+K]^+^	Phosphatidylcholine (C16∶0/C22∶6)
866.65	866.67	0.02	[M+K]^+^	Glucosylceramide (d18∶1/C24∶0h)
869.67	869.69	0.02	[M+K]^+^	Triacylglycerol (C16∶0/C16∶0/C18∶2)
880.62	880.69	0.07	[M+K]^+^	Glucosylceramide (d18∶1/C26∶1)
895.64	893.71	0.07	[M+K]^+^	Triacylglycerol (C16∶0/C18∶2/C18∶1)
894.65	894.67	0.02	[M+K]^+^	Glucosylceramide (d18∶1/C26∶0h), (t18∶1/C26∶1)
897.72	897.73	0.01	[M+K]^+^	Triacylglycerol (C16∶0/C18∶1/C18∶1)
906.63	906.7	0.07	[M+K]^+^	Glucosylceramide (d18∶1/C28∶0)

Underlined molecules are only predicted molecules based on the localization and molecular mass.

### Imaging Mass Spectrometric Analysis of Ceramide Precursors in Murine Footpad Skin

Both GlcCer and SM, which are immediate precursors that generate Cer in the SC, are converted to Cer during transition from SG/SS to SC in epidermis. The abnormality of these conversion has been shown in a pathological condition, such as hyperplasia, as well as inherited disease, *i.e.,* deficiencies of ß-glucoserebrosidase, activator protein of ß-glucoserebrosidase (sapocin C), or sphingomyelinase [40]. Therefore, assessments of GlcCer and SM are a diagnostic approach to these conditions. IMS analysis revealed the presence of at least three SM species (*m/z* 713.49, 741.51, and 769.52) (Fig. 3j, k, l) and four predictable GlcCer possible ions (*m/z* 866.65, 880.62 894.65, and 906.63) (Fig. 3p, q, r, s). For tandem mass spectrometric analyses of SM, we detected the neutral loss (NL) of 53 and 183 Da that corresponds to the trimethylamine [(CH3)3N] and the head group [(CH3)3N(CH2)2PO4H] of SM [41] and the fragment ion of d18∶1 (*m/z* 305.0) (Fig. S3) [42], identifying these three as SM species (d18∶1/C14∶0, d18∶1/C16∶0, and d18∶1/C18∶0) (Table 1). Since similar to Cer, GlcCer is a difficult molecular species to ionize compared with other lipid species, to further increase the sensitivity of GlcCer, we minimized the mass range (*m/z* 800–1200) to allow the efficient accumulation of these ions into the quadrupole ion trap, and succeeded to get clear localization of GlcCers and reproducibility (Fig. 3, tu, v, w and Fig. S4). Tandem mass spectrometric analysis showed the neutral loss (NL) of glucose from a precursor ion and assigned *m/z* 732.6 as a (d18∶1/C26∶0h), while tandem mass spectrometric analyses of other GlcCer species were not completed due to low signal intensities/sensitivity issues. Therefore, we deduced these ions as GlcCer possible species, *i.e.,* GlcCer (d18∶1/C24∶0h, d18∶1/C26∶1, d18∶1/C26∶0h, and d18∶1/C28∶0) (Table 1).

All SM species were distributed throughout epidermis and dermis. On the other hand, GlcCer, including GlcCer possible ions, were localized at epidermis specifically. In addition, interestingly the composition of FA is completely different between SM and GlcCer (Table 1).

### Phosphatidylcholine Detection by Imaging Mass Spectrometry

As shown in Figure 1b, PCs are also major epidermal lipid species in the mouse footpad. IMS analysis shows that three molecular species of PCs, a major component of cellular membrane species, are localized across the skin, including the dermis and significantly decreased (or at trace levels) in the SC (Fig. 3m, n, o). These molecules are annotated based on previous reports [6] [43] and tandem mass spectrometric analyses. Tandem mass spectrometric analysis showed the NL of head group and fatty acids, respectively, and assigned molecular species (Fig. S3 and Table 1). Notably, the molecular ion at *m/z* 796.5 assigned as PC (diacyl 16∶0–18∶2) was detected epidermis main PC species, while *m/z* 798.5 assigned as PC (diacyl 16∶0–18∶1) was detected in both epidermis and dermis. Since PC is a major membrane constituent, membrane property likely differs in keratinocytes and fibroblasts, and it mainly constituted in epidermis and dermis, respectively.

### Clinical Application of Imaging Mass Spectrometric Analyses

**Figure 4:**
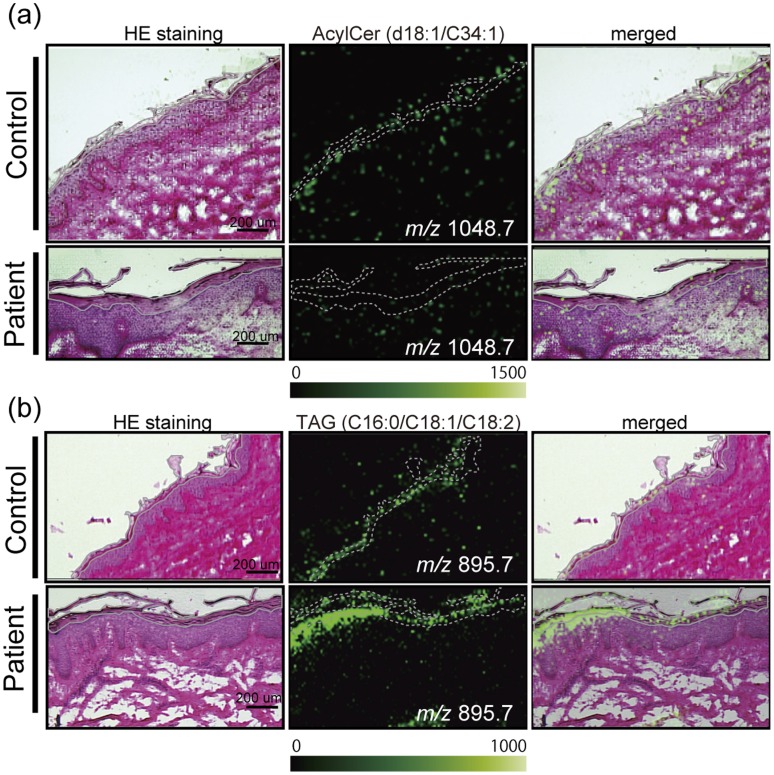
IMS analyses of clinical samples. IMS analysis demonstrates deficiencies of AcylCer (a) (S/N ratio: 9) and, conversely, the accumulation of TAG (b) in the SC (indicated as a gray line) of a DCS patient compared with a normal human subject. The scale bar shows 200 µm. The imaging pixel is 10 µm. Color bar shows signal intensity.

We recently found that AcylCer deficiencies occur in Dorfman–Chanarin syndrome (DCS), an autosomal recessive, neutral lipid storage disorder with ichthyosis, due to loss-of-function mutations in CGI-58 (α/β-hydrolase domain containing protein 5, ABHD5) in human skin [44]. Therefore, we performed IMS analyses of these skins for clinical application to assess whether TAG accumulation, including linoleate-containing species and AcylCer deficiencies, occur in the SC of DCS. To further increase the sensitivity of IMS, we minimized the mass range (*m/z* 800–1200 for TAG, and *m/z* 900–1200 for AcylCer) to allow the efficient accumulation of these ions into the quadrupole ion trap and to prevent ion suppression by other lipids. IMS analysis demonstrated that the ion signals of possible AcylCer were significantly attenuated, and conversely that TAGs accumulated in the SC of the patient compared with the control (Fig. 4). Consistent with our prior lipid analysis of lipid extracts from control normal SC [44], the AcylCer-related ion (*m/z* 1048.7, d18∶1/C34∶1) was found to be present in the control normal SC. The AcylCer signal had only trace-level intensity in the patient (Fig. 4a). Moreover, tandem mass spectrometric analysis proved that the accumulation of TAG containing linoleate (*m/z* 895.7, C16∶0/C18∶2/C18∶1) is one of the major molecular species in the SG, where AcylCer is synthesized, and is also retained in the SC (Fig. 4b). These results clarified that AcylCer deficiencies in the SC of DCS are caused by decreases of that synthesis, rather than misslocalization in the epidermis. Moreover, TAG accumulation does not significantly occur in extra SC regions in skin.

## Discussion

TLC analysis of extracted lipids suggests that Cers (amide-linked non-hydroxy fatty acid and sphingosine) were a primary target in the mouse footpad epidermis by IMS analysis because the relative amounts of Cer species were higher than the other lipids (Fig. 1). Since a previous report described that the AP ion source exhibits soft ionization [45], we compared ionization patterns of Cer between our AP ion-source instrument and vacuum-type instrument. We found that although the S/N ratio of the two instruments is nearly equal, the ionization tendency of the AP ion-source instrument is different from that of a vacuum-type instrument. As shown in Figure 2, the AP ion-source instrument demonstrated a simple ionization pattern of Cer; but the vacuum type showed multiple adduct ions, which made it complicated. Therefore, our developed instrument equipped with an AP ion-source chamber, which shows the most intense peak with the Cer [M−H_2_O+H]^+^ion, is suitable for ionization of Cer.

Next, we deposited the Cer standard onto a mouse brain tissue section to validate the ionization pattern on tissue sections. Three tissue-derived signals (*m/z* 638.9, 772.5, and 798.5) were thoroughly distributed in the brain sections. The background signals derived from matrix-related peaks (*i.e.,* signals outside of the tissue) had a one or zero at the first decimal point, and the target lipid derived signals that we assigned showed 4 to 6 at the first decimal point. The mass tolerance of our machine was lower than 0.1 Da in the range of *m/z* 500–1200. Therefore, it is unlikely that lipid-derived signals overlapped with matrix signals. In contrast, the ion image at *m/z* 632.5 showed high intensity on the deposited area, revealing that the *m/z* 632.5 was exogenous Cer. Moreover, the ion image at *m/z* 632.7 showed a completely different image than *m/z* 632.5, meaning we could distinguish these ions individually (Fig. 2h). The mass range around 600–800 is complicated with various molecules especially with a vacuum-type instrument, but not with an AP ion-source instrument (Fig. 2c, d). Because Cer display molecular heterogeneity in certain tissues, in particular epidermis, a simplified spectrum to distinguish their mass is an important requirement. These results suggest that IMS with AP ion-source instrument is appropriate to analyze Cer species on tissues.

Previous immunoelectron microscopic analysis using an anti-Cer antibody showed that anti-Cer staining on the human epidermis was abundantly concentrated at the cell membranes and/or intercellular space in the SC and in the perinuclear region of the cells in the lower SS [38]. A recent report of skin IMS demonstrated the presence of Cer1P (Cer has not been reported) [36]. However, the spatial resolution was not sufficient (30–150 µm), and plural molecular species have not yet been detected. We demonstrated here the distribution of various kinds of Cer molecular species, as well as Cer1P on the footpad skin by IMS (Fig. 3). All Cers were enriched at SC and SG (white-line area). These findings are consistent with the previous study by immunoelectron microscopic analysis [38], as well as lipid quantification from each epidermal layer [46], showing that the Cer content is high at differentiated layers of the epidermis, SC, SG and SS. We summarized the deduced molecular structures based on the previous report [36] compared with the theoretical mass (Table 1). Moreover, we also demonstrated a different localization of Cer-related species due to their amide-linked fatty acid composition (Fig. S2). The result suggested that each Cer-related species shows a different distribution, according to its fatty acid composition.

In the epidermis, some pools of GlcCer and SM localized in the SG are immediate precursors of Cer in the epidermis. As shown in Figure 3, GlcCer was fairly well detected in the brown-line area (SG and SS) but not in the dermis, while SM levels were the same throughout the epidermis and dermis. These results are consistent with prior biochemical analysis showing that GlcCer, but not SM, levels significantly increase during epidermal differentiation [47]. Importantly, our IMS analysis showed that the distribution of amide-linked fatty acid of GlcCer and SM species are C24–28 and C16–18, respectively, while the major epidermal Cer is C22–32 in SC, as elucidated by IMS (Table 1), suggesting that GlcCer rather than SM appears to the major pool for Cer species production in the SC (Fig. S1). These results are comparable with a previous study, which detected Cer but not GlcCer in the SC layers.

Out studies reveal that PCs are one of the major lipids in skins and are localized using IMS. Not only Cers, but also PC molecular species shows different signal patterns due to the FA composition. Consistent with prior studies analyzing lipid extracts of epidermal fractions, PC is trace (or not preset) in the SC, further validating the IMS alternative method.

We recently found that AcylCer deficiencies occur in DCS, due to loss-of-function mutations in CGI-58 in human skin [44]. In addition, *Cgi-58 null* mice display the same features, *i.e.,* lack of AcylCer in parallel with epidermal permeability barrier defects [48]. CGI-58 is a cofactor of adipose TAG lipase and other still unidentified TAG lipases. Indeed, oil red O-stained lipid droplets are present within keratinocytes in the SC, SG, and SB [48]. Because AcylCers are required for normal permeability barrier formation, AcylCer deficiencies most likely contribute to the barrier abnormalities in DCS [44], in addition to lamellar/nonlamellar phase separation due to accumulated TAG within the extracellular domains of the SC [49]. It has been suggested that linoleate from TAG is preferentially utilized for the acylation of ω-hydroxyCer to generate AcylCer. In this study, we showed that AcylCer deficiencies do occur at both the SG and SC, eliminating a possibility of misslocalization of AcylCer. As shown in Figure 4, the ion signals relating to AcylCer are significantly attenuated; on the other hand, TAGs were accumulated in the SC of DCS patients compared with the control. These results not only proved our previous findings, *i.e.,* the accumulation of TAG and deficiencies of AcylCer, but also demonstrated the distribution of AcylCer and TAG, their molecular species, and their abnormalities that likely contribute to permeability barrier defects in patient skin.

This is the first report to describe Cer imaging by MALDI-IMS and also to characterize the distribution of Cer species within the epidermal layers of clinical samples using our developed machine, which has an AP ion-source. We were able to obtain detailed distributions of Cers including Cer1P, AcylCer, GlcCer, as well as SM, PC, and TAG in murine and human skin. In contrast to conventional lipid analysis using lipid extracts, which requires large amounts of samples, IMS allows Cer analysis using 3–5 mm diameter biopsy samples. Therefore, IMS can minimize a limitation to assessing lipid profiles in cells/tissues, in particular human samples. IMS is a useful method for diagnosing lipid metabolic abnormalities in several cutaneous diseases and for investigating their pathogeneses that can lead to developing new therapeutic approaches. In summary, our study has illuminated a novel approach for investigating roles of Cer and other lipids in skin and/or diagnosis of diseases, as well as for further application of IMS technology in biomedical fields. Moreover, the IMS apparatus of an AP-MALDI and a QIT-TOF mass spectrometer, which output simple spectrum, is useful for imaging heterogeneous Cer species in cells and tissues.

## Materials and Methods

### Ethics Statement

For human samples, informed consent was obtained from all volunteers before participation. Subjects consented in written form to cooperate after they were informed. This study was specifically approved by the Institute Ethical Review Board of the Hamamatsu University School of Medicine, and performed according to the Declaration of Helsinki Principles. For animal samples, all experiments in this study were specifically approved by the Ethics Committee at the Hamamatsu University School of Medicine. And all efforts were made to minimize suffering.

### Reagents and Materials

All solvents used for MS analyses were of HPLC grade and were purchased from Kanto Chemical Co., Inc. (Tokyo, Japan). Bradykinin and angiotensin-II were obtained from Sigma-Aldrich Japan (Tokyo, Japan) and used as calibration standards. 2, 5-Dihydroxy benzoic acid (DHB) was used as the matrix (Bruker Daltonics, Leipzig, Germany). Sodium carboxymethyl cellulose (CMC) was from Wako Pure Industries LTD (Osaka, Japan). C57BL/6Cr mice were from Japan SLC (Shizuoka, Japan). *Cer and PC standards were purchased from Toronto Research Chemicals Inc. (North York, Canada) and* Avanti Polar Lipids (Alabaster, AL, USA), respectively.

### Clinical Samples

DCS patients’ skin was obtained as described previously [44]. Briefly, collected tissues were embedded in 2% pre-cooled *CMC and were then* sectioned to a 10-µm thickness at −20°C using a Leica CM1950 cryostat (Leica *Microsystems*, Wetzlar, *Germany).* The control skin was gifted from a volunteer without any skin diseases. We made three sections per person to get reproducibility of measurements.

### Tissue Preparation

We used the footpad skin of 8-week-old C57BL/6Cr mice from Japan SLC (Shizuoka, Japan). The tissues were embedded in 2% pre-cooled *CMC and were then* sectioned to a 10-µm thickness at −20°C using a Leica CM1950 cryostat (Leica *Microsystems*, Wetzlar, *Germany). The brain tissues were snap-frozen in liquid nitrogen directly and sectioned to a 10-*µm thickness. Frozen sections were thaw-mounted on indium-tin-oxide (ITO)-coated glass slides (Bruker Daltonics). We used a DHB solution (50 mg/mL in 70% methanol) as the matrix. The matrix solution was uniformly sprayed over the tissue surface using a 0.2-mm nozzle-caliber air brush (Procon Boy FWA Platinum; Mr. Hobby, Tokyo, Japan). Continuous frozen sections were also thaw-mounted on MAS-coated glass slides (Matsunami Glass Industries, Ltd., Osaka, Japan) for conventional hematoxylin-eosin (HE) staining. One µg of Cer standard was deposited on a brain section.

### Thin-layer Chromatography

We used the footpad skin of 8-week-old C57BL/6Cr mice from Japan SLC (Shizuoka, Japan). Epidermal lipids were extracted from murine tissue using a 20-fold volume of solvent, incubated twice with chloroform/methanol (2∶1, v/v) overnight at room temperature. Lipids were separated on a silica gel 60 HPTLC plates (Merck, Darmstadt, Germany) using methylacetate/propanol/chloroform/methanol/0.25% aqueous CaCl_2_, (25/25/25/10/9, v/v/v/v) for separation of phospholipids. A four-sequence solvent system was used to isolate Cer species: 1) chloroform/methanol/water (40/10/1, v/v/v) 1.8 cm, 2) chloroform/methanol/water (40/10/1, v/v/v) 4.5 cm, 3) chloroform/methanol/acetic acid (47/2/0.5, v/v/v) 8.5 cm, and 4) n-hexane/diethylether/acetic acid (30/10/0.5, v/v/v) 8.5 cm. Lipids were visualized using 0.1% primuline reagent under UV light at 365 nm.

### Mass Spectrometry

MALDI-TOF mass spectrometric analyses of vacuum-MALDI were performed using a MALDI-hybrid quadrupole TOF-type mass spectrometer (QSTAR Elite, AB Sciex, Foster City, CA) equipped with an orthogonal MALDI source and an Nd:YAG laser at a repetition rate of 200 Hz. Samples were analyzed in positive ion mode over the range of *m/z* 400–1000. The mass spectra were calibrated externally using a standard peptide calibration mixture containing 10 pmol/*µ*l each of bradykinin peptide fragment (amino acid residue 1–7) ([M+H]^+^, *m/z* 757.4) and human angiotensin-II peptide fragment ([M+H]^+^, *m/z* 1046.5). A methanol/0.1% TFA solution (1/1, v/v) containing 10 mg/ml DHB was used as the matrix. For comparison with AP ion-source mass spectrometer, serial sections (n = 3) were prepared and were matrix sprayed at the same time. The spectra were extracted from same positions.

### Imaging Mass Spectrometry

IMS analyses were performed by an AP ion-source mass spectrometer with a laser frequency of 1000 Hz (laser diameter of 10 µm). All analyses were performed in the positive-ion mode within the mass ranges of *m/z* 600–1200 for Cer, Cer1P, PC, and SM, *m/z* 800–1200 for TAG and GlcCer, and *m/z* 900–1200 for AcylCer. A 10-µm raster width was set to generate images of the skin, and a 50-µm raster width was set to generate the images of the brain. The ion images were constructed using BioMap software (Novartis, Basel, Switzerland). Embedded compounds are required to prepare skin section. We used 2% pre-cooled *CMC* for embedding compounds. Levels of signal on outside of the section, which is derived from CMC, were employed as the threshold, i.e., 150 to 500 for Cer1P, Cer, SM, and PC, and 50–200 for GlcCer. All spectra were normalized by total ion current.

## Supporting Information

Figure S1 **Generation of ceramide in stratum corneum.** Abbreviations for Cer structures are according to (Motta et al., Biochim Biophys Acta 1182∶147-151, 1993 and Robson et al., J Lipid Res 35∶2060-2068,1994). N, A and EO indicate amide-linked fatty acid (FA) species: N, non-OH FA; A, 2-OH FA; EO,omega-O-esterified FA. S, sphingosine; P, phytosphingosine (or 4-hydoxysphinganine); H, 6-hydroxysphingosine indicate sphingosine base structures. Cer 2 (NS) are ubiquitously expressed in mammalian tissues, while late stages of differentiation produce heterogeneous Cer species. In particular, Cer 1 (EOS), Cer 4 (EOH) and Cer 9 (EOP) are unique to the epidermis.
(TIF)

Figure S2 **Mass Microscope characterizes the different distribution of Cer Species in murine skin.** The merged image of three Cer1P ion images shows their different distributions. We selected three Cer1P molecular species at *m/z* 618.4, 646.4, and 730.5 suggesting Cer1P (d18∶1/C16∶0), Cer1P (d18∶1/C18∶0), and Cer1P (d18∶1/C24∶0), respectively. The ion images at *m/z* 618.4 and *m/z* 646.4 were detected in the middle of SC regions, while the ion image at *m/z* 730.5 was detected in relatively lower SC. Scale bar showed 200 µm.
(TIF)

Figure S3 **Tandem mass spectrometric analyses on tissue sections.** Tandem mass spectrometric analyses of Cer, Cer1P, SM, PC, TAG and GlcCer were performed on tissue sections. (a) Tandem mass spectrum of *m/z* 646.4. (b) Tandem mass spectrum of *m/z* 630.5. The spectra (c) and (d) were standard mass spectrum of Cer1P and Cer, respectively. The fragment patterns of these spectra were compared and confirmed the structure. The tandem mass spectrometric analyses of representative SM (e), PC (f), TAG (g) and GlcCer (h) were also performed and confirmed their structures.
(TIF)

Figure S4 **GlcCer distribution of other murine footpad sections.** The signal intensity of GlcCer is very low and hard to get tandem mass spectrometric data. Therefore, we minimize the *m/z* range to concentrate these molecules in quadrupole ion trap. We made multiple sections and get reproducibility of these moelcular localization. As described, the signals are predominantly detected in SS and SG regions (white-line area). Lower pannels show the statistical analyses results of ion signal intensity between biological regions and out of sections. As shown in bar graph, the significant difference tendencis are existed (SM and PC: p<0.05, GlcCer: p<0.1).
(TIF)
