# Supplementary figures and images for: Imaging Mass Spectrometry Visualizes Ceramides and the Pathogenesis of Dorfman-Chanarin Syndrome Due to Ceramide Metabolic Abnormality in the Skin
Source: PLoS One. 2012 Nov 15;7(11):e49519. doi: 10.1371/journal.pone.0049519 (PMC3499467; doi:10.1371/journal.pone.0049519)

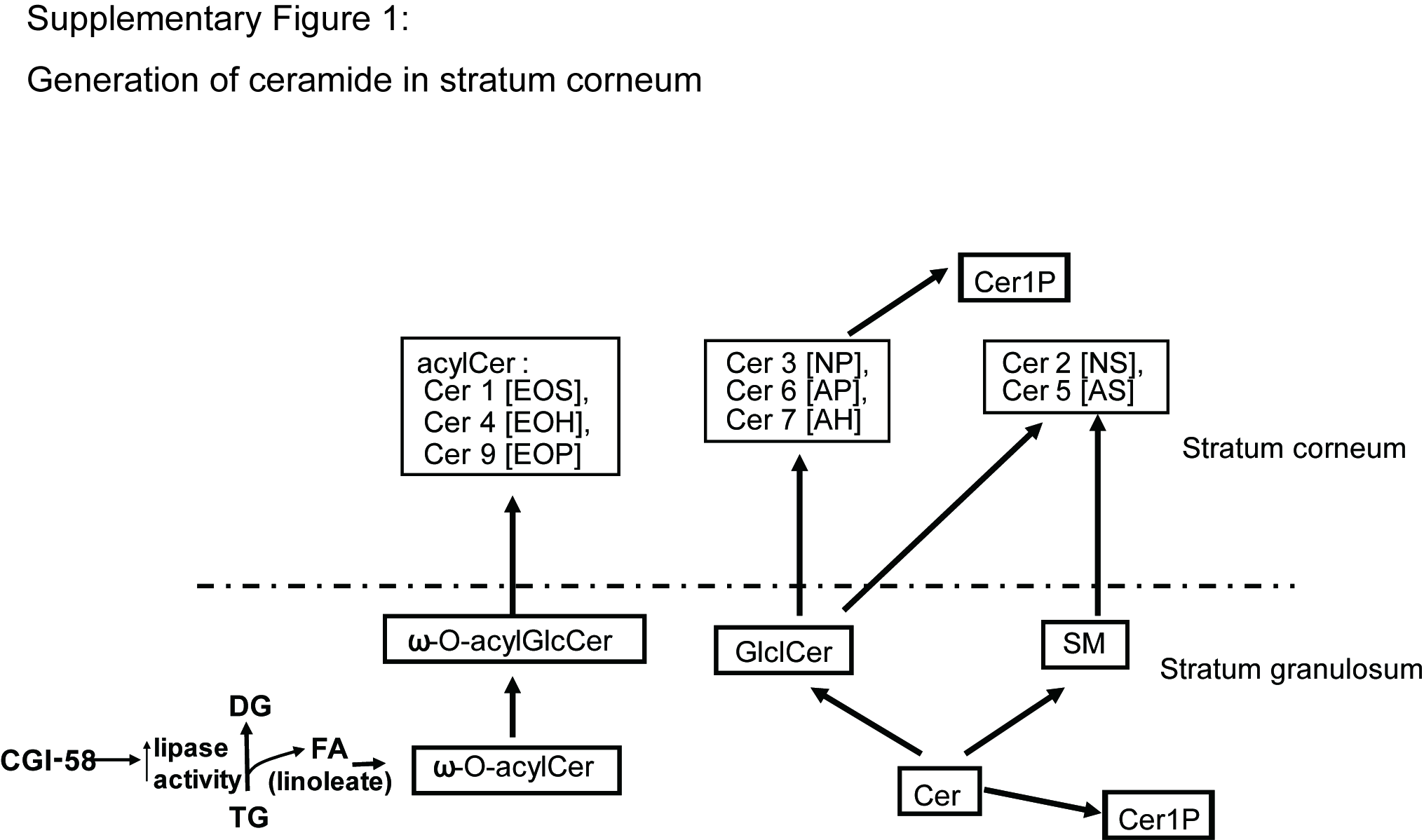

Supplement: Figure S1 — Generation of ceramide in stratum corneum. Abbreviations for Cer structures are according to (Motta et al., Biochim Biophys Acta 1182∶147-151, 1993 and Robson et al., J Lipid Res 35∶2060-2068,1994). N, A and EO indicate amide-linked fatty acid (FA) species: N, non-OH FA; A, 2-OH FA; EO,omega-O-esterified FA. S, sphingosine; P, phytosphingosine (or 4-hydoxysphinganine); H, 6-hydroxysphingosine indicate sphingosine base structures. Cer 2 (NS) are ubiquitously expressed in mammalian tissues, while late stages of differentiation produce heterogeneous Cer species. In particular, Cer 1 (EOS), Cer 4 (EOH) and Cer 9 (EOP) are unique to the epidermis. (TIF) [file pone.0049519.s001.tif]

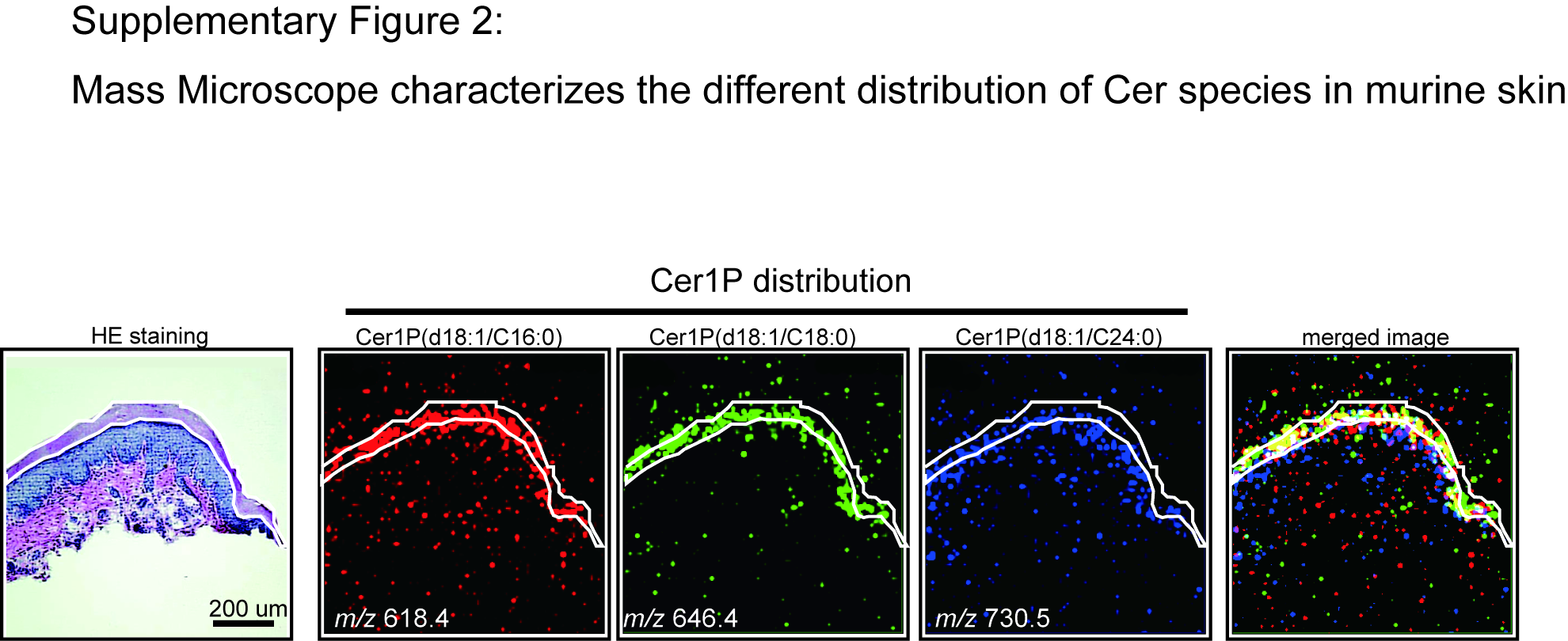

Supplement: Figure S2 — Mass Microscope characterizes the different distribution of Cer Species in murine skin. The merged image of three Cer1P ion images shows their different distributions. We selected three Cer1P molecular species at m/z 618.4, 646.4, and 730.5 suggesting Cer1P (d18∶1/C16∶0), Cer1P (d18∶1/C18∶0), and Cer1P (d18∶1/C24∶0), respectively. The ion images at m/z 618.4 and m/z 646.4 were detected in the middle of SC regions, while the ion image at m/z 730.5 was detected in relatively lower SC. Scale bar showed 200 µm. (TIF) [file pone.0049519.s002.tif]

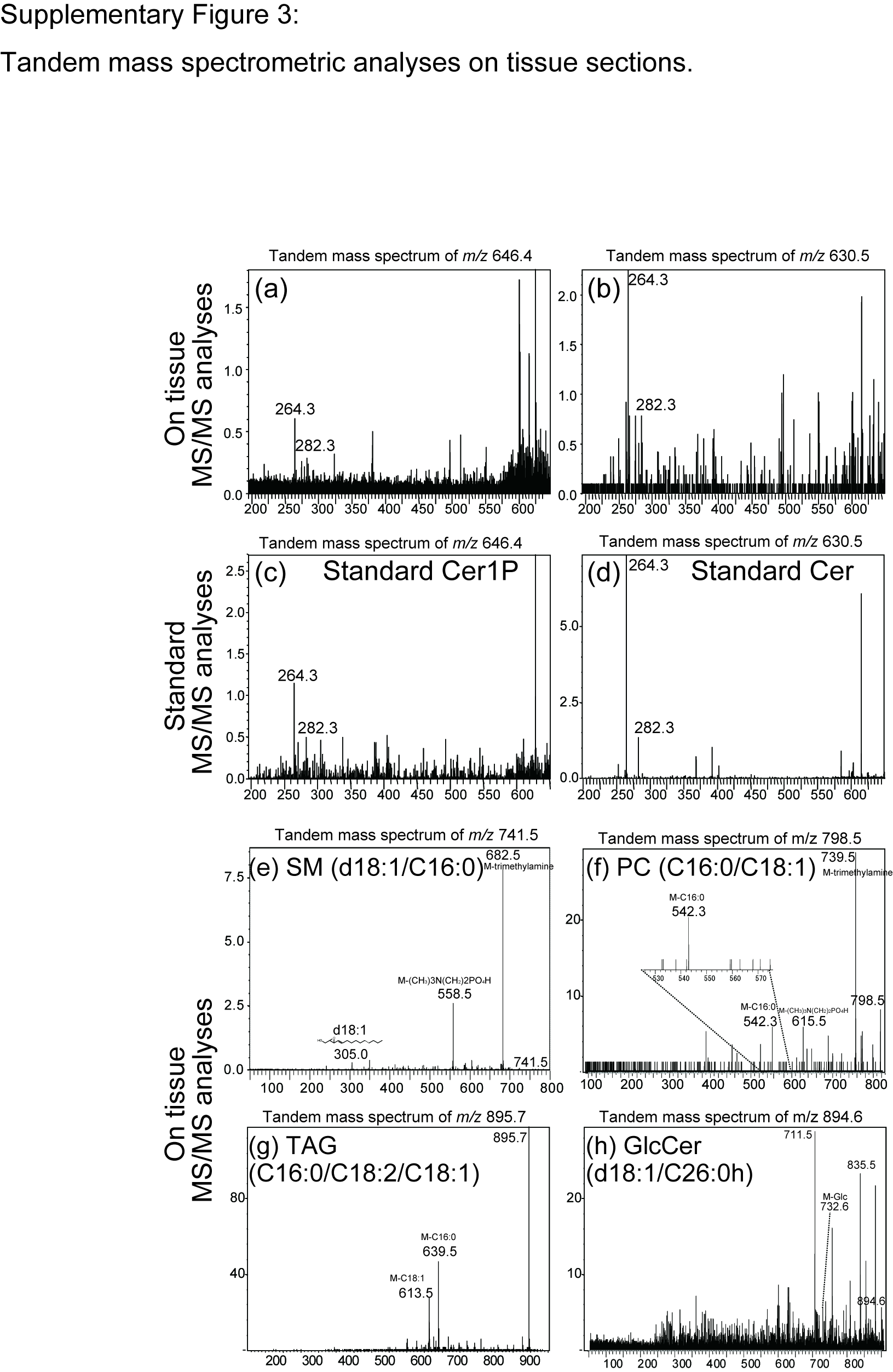

Supplement: Figure S3 — Tandem mass spectrometric analyses on tissue sections. Tandem mass spectrometric analyses of Cer, Cer1P, SM, PC, TAG and GlcCer were performed on tissue sections. (a) Tandem mass spectrum of m/z 646.4. (b) Tandem mass spectrum of m/z 630.5. The spectra (c) and (d) were standard mass spectrum of Cer1P and Cer, respectively. The fragment patterns of these spectra were compared and confirmed the structure. The tandem mass spectrometric analyses of representative SM (e), PC (f), TAG (g) and GlcCer (h) were also performed and confirmed their structures. (TIF) [file pone.0049519.s003.tif]

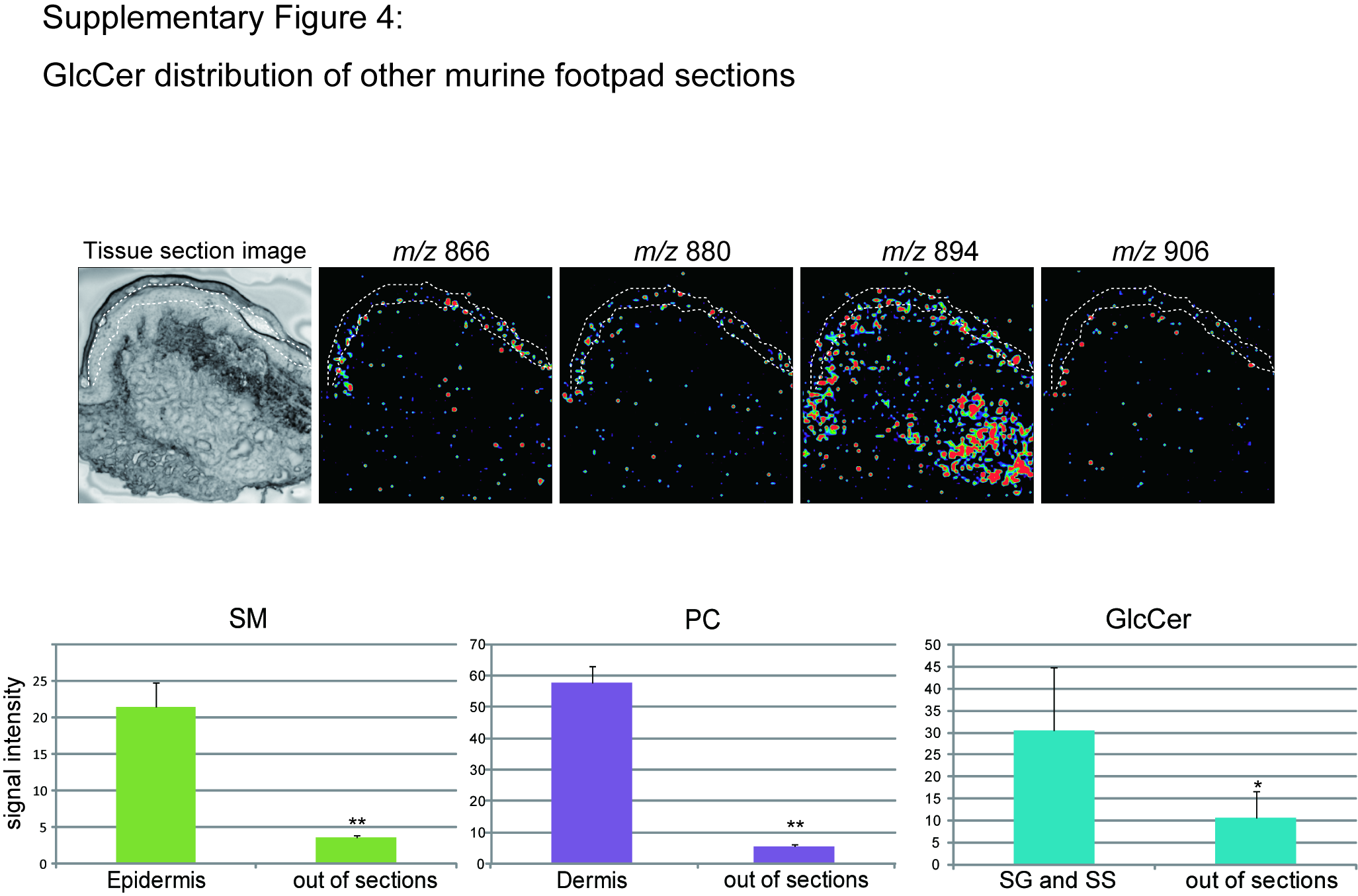

Supplement: Figure S4 — GlcCer distribution of other murine footpad sections. The signal intensity of GlcCer is very low and hard to get tandem mass spectrometric data. Therefore, we minimize the m/z range to concentrate these molecules in quadrupole ion trap. We made multiple sections and get reproducibility of these moelcular localization. As described, the signals are predominantly detected in SS and SG regions (white-line area). Lower pannels show the statistical analyses results of ion signal intensity between biological regions and out of sections. As shown in bar graph, the significant difference tendencis are existed (SM and PC: p<0.05, GlcCer: p<0.1). (TIF) [file pone.0049519.s004.tif]
